# Navigating the Diagnostic and Therapeutic Challenges of Central Neurocytoma: A Case Report

**DOI:** 10.7759/cureus.60882

**Published:** 2024-05-22

**Authors:** Soumya Agrawal, Samarth Shukla, Sunita Vagha

**Affiliations:** 1 Pathology, Jawaharlal Nehru Medical College, Datta Meghe Institute of Higher Education and Research, Wardha, IND

**Keywords:** neurological deficits, multidisciplinary management, surgical intervention, hydrocephalus, intracranial tumor, central neurocytoma

## Abstract

Central neurocytoma, a rare intracranial tumor predominantly located in the lateral and third ventricles, presents a diagnostic and therapeutic challenge due to its varied clinical manifestations. We report the case of a 53-year-old male presenting with right upper and lower limb weakness, headaches, blurred vision, and tingling sensations, leading to the diagnosis of central neurocytoma with associated hydrocephalus. Initial evaluation, including magnetic resonance imaging (MRI) and subsequent computed tomography (CT) scans, revealed characteristic features of the tumor. The patient underwent a two-stage surgical intervention, including tumor excision and ventriculoperitoneal shunting, followed by a tracheostomy due to respiratory complications post-surgery. Histopathological examination confirmed the diagnosis of central neurocytoma, prompting multidisciplinary management and further referral for long-term follow-up. This case underscores the importance of comprehensive evaluation, multidisciplinary collaboration, and continued research in optimizing the diagnosis and management of central neurocytomas.

## Introduction

Central neurocytoma is a rare intracranial tumor primarily located in the lateral and third ventricles of the brain. First described by Hassoun and colleagues in 1982, central neurocytoma typically affects young adults, with a peak incidence in the third decade of life [1]. These tumors are histologically characterized by uniform round cells with neuronal differentiation. They are classified as grade II or IV according to the World Health Organization's (WHO) classification of central nervous system tumors [2]. The clinical presentation of central neurocytoma varies widely and may include symptoms related to increased intracranial pressure, such as headache, nausea, vomiting, and papilledema. Focal neurological deficits such as hemiparesis, visual disturbances, and sensory abnormalities may also occur, depending on the location and size of the tumor [3].

The diagnosis of central neurocytoma typically relies on neuroimaging modalities such as magnetic resonance imaging (MRI) and computed tomography (CT) scans, which reveal a well-circumscribed intraventricular mass with characteristic imaging features, including iso- to hyperintense signal on T1-weighted MRI and heterogeneous enhancement following contrast administration [4]. Treatment of central neurocytoma often involves surgical resection of the tumor to relieve symptoms and prevent complications such as obstructive hydrocephalus. Gross total resection is associated with favorable outcomes, although subtotal resection may be necessary in cases where complete excision is not feasible due to the proximity to critical neurovascular structures [5]. Despite advances in neurosurgical techniques, central neurocytoma remains a challenging entity due to its rarity and variable clinical presentation. Multidisciplinary management involving neurosurgeons, neuroradiologists, and neuropathologists is essential for optimal patient care and long-term outcomes.

## Case presentation

A 53-year-old male presented to the outpatient department of the super-speciality hospital with a myriad of symptoms: right upper and lower limb weakness persisting for two months, headaches ongoing for 15 days, bilateral blurring of vision also for 15 days, and tingling sensation in the right upper and lower limbs for the past eight days. His medical history revealed a prior hospitalization seven years ago for mental illness, for which he was not on medication.

**Figure 1:**
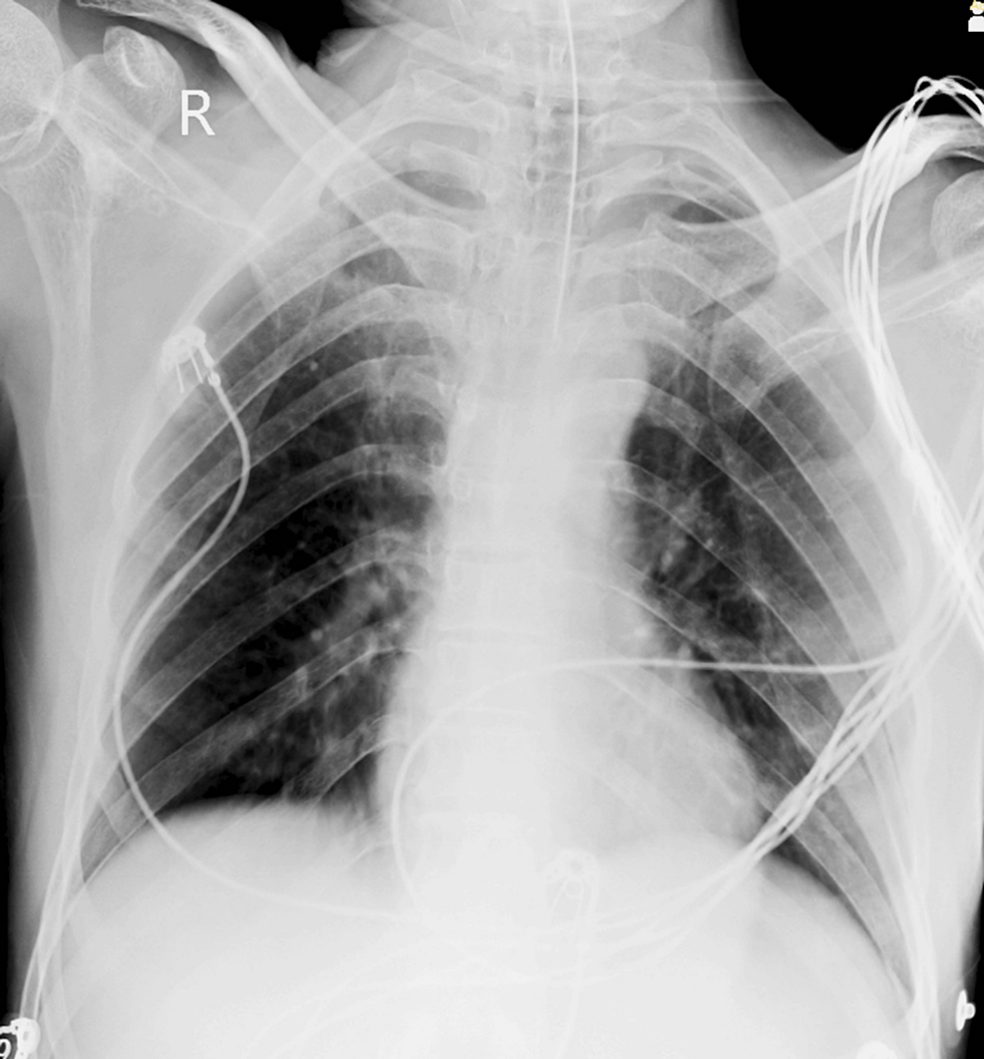
The chest X-ray of the patient showed normal findings.

**Figure 2:**
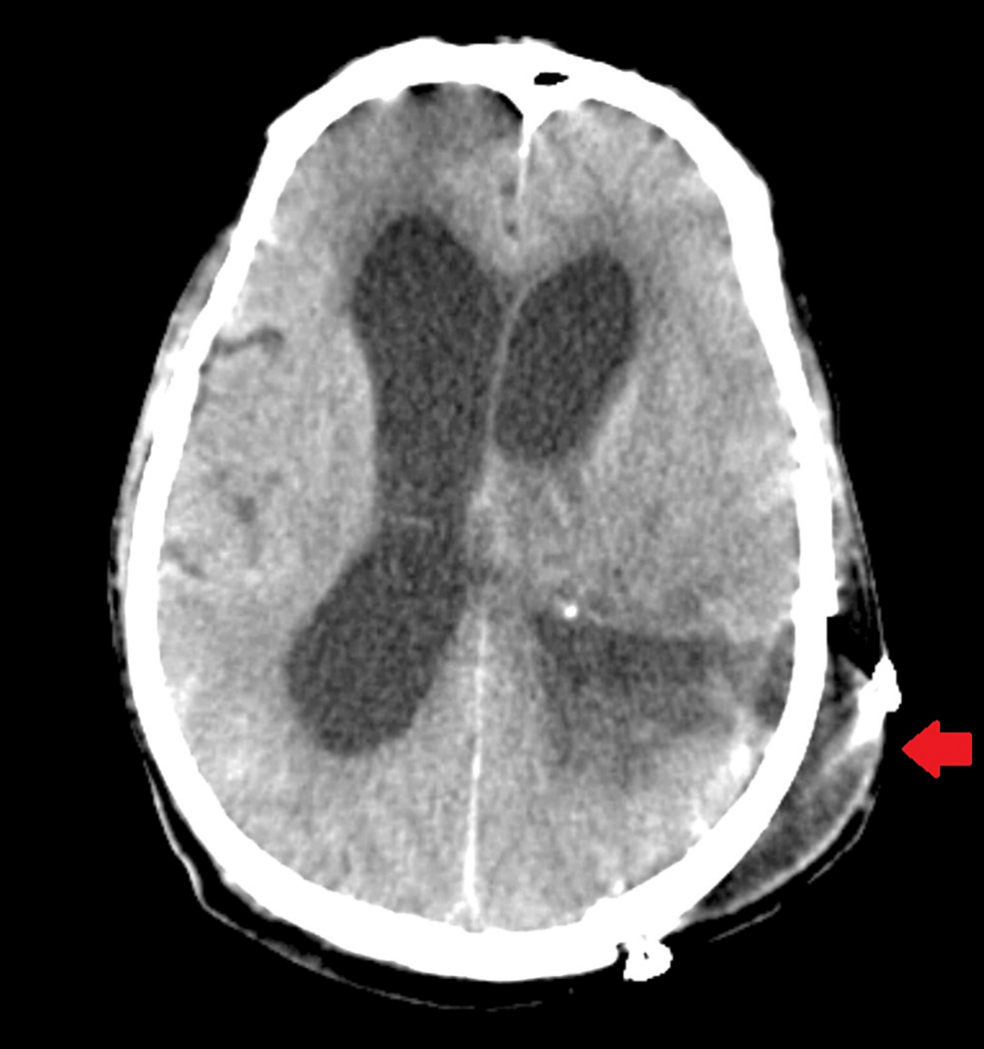
The head CT of the patient revealed bilateral dilatation and communicating hydrocephalus, along with evidence of a brain tumor.

Upon examination, his respiratory system appeared normal with bilateral air entry, his cardiovascular system exhibited regular S1 and S2 sounds, and his central nervous system showed a Glasgow Coma Scale score of E4, V5, and M6, with bilateral reactive pupils to light. Local examination revealed weakness in the right upper and lower limbs. Initial blood tests, including a complete blood count, liver function test, kidney function test, and chest X-rays, yielded normal results (Figure 1), prompting a recommendation for an MRI of the brain. Subsequent MRI results indicated features consistent with intense characteristics and a mass effect, most likely indicative of a meningioma. However, other rare possibilities, such as a vein of Galen thrombosis, were considered satisfactory. Following this, the patient developed hydrocephalus. CT scans of the head revealed bilateral dilatation and communicating hydrocephalus, along with evidence of a brain tumor (Figure 2). Consequently, the patient was diagnosed with a brain tumor after a thorough discussion among the healthcare team members, leading to a decision to proceed with surgery. Informed consent was obtained from the patient and their relatives after counseling.

**Figure 3:**
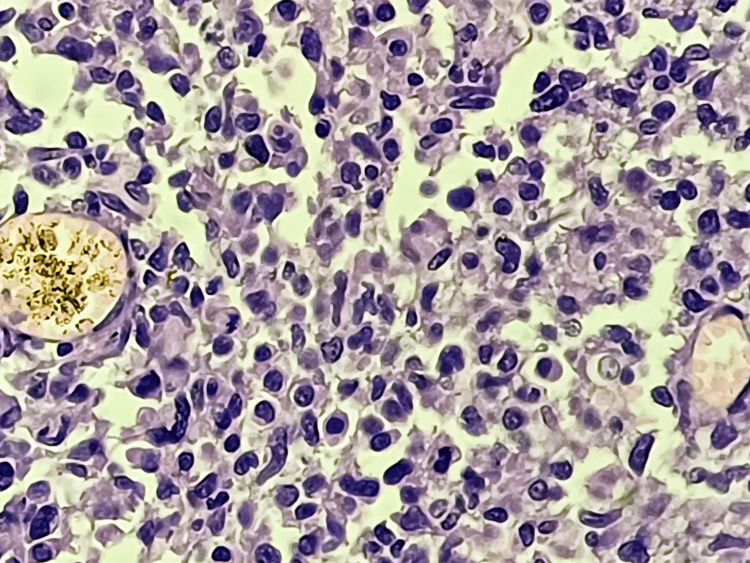
Central neurocytoma at 40x: The section showed densely populated small, monomorphic cells embedded in a variable, abundant, and delicate fibrillar matrix. Rounded nuclear contours, perinuclear clearing, a plexiform capillary arcade, and associated microcalcifications impart an oligodendroglioma-like appearance.

The first surgical procedure involved a left parietal occipital craniotomy for tumor excision under general anesthesia. This procedure entailed a left parieto-occipital skin incision, burr hole creation, durotomy, and tumor dissection, followed by closure in layers. The second procedure, ventriculoperitoneal (VP) shunting, also performed under general anesthesia, involved positioning the patient supine with the head turned to the left. An incision above the pinna and another in the abdomen facilitated the shunt insertion, connecting the ventricle to the peritoneum to alleviate hydrocephalus. Due to difficulty maintaining oxygen saturation on room air post-surgery, oxygen administration proved insufficient, necessitating a planned tracheostomy as the third procedure. This involved an incision above the sternal notch, followed by dissection to expose the trachea and subsequent insertion of a tracheostomy tube. Histopathological examination confirmed a central neurocytoma (WHO grade II or IV) (Figure 3), prompting referral to the neurosurgery department for further management.

## Discussion

The presented case underscores the diagnostic and therapeutic challenges associated with central neurocytoma, a rare intracranial tumor with diverse clinical manifestations. In this case, the patient presented with a constellation of symptoms, including right upper and lower limb weakness, headaches, bilateral blurring of vision, and tingling sensations suggestive of central nervous system involvement. These symptoms prompted further investigation, leading to the discovery of an intraventricular mass consistent with central neurocytoma. The clinical presentation in our case aligns with previous reports highlighting the variability in symptoms associated with central neurocytoma, emphasizing the importance of considering this diagnosis in patients presenting with neurological deficits and hydrocephalus [6,7]. Neuroimaging, particularly MRI and CT scans, is pivotal in diagnosing and characterizing central neurocytoma. In this case, MRI revealed characteristic features of the tumor, including intense characteristics and mass effect, prompting further evaluation and surgical intervention. The imaging findings corroborate existing literature, which describes central neurocytoma as a well-circumscribed intraventricular mass with iso- to hyperintense signal on T1-weighted MRI and heterogeneous enhancement following contrast administration [8,9].

Surgical resection remains the cornerstone of treatment for central neurocytoma, aiming to alleviate symptoms, prevent complications such as obstructive hydrocephalus, and achieve long-term disease control. In our case, the patient underwent two surgical procedures: tumor excision followed by ventriculoperitoneal shunting to address hydrocephalus. The decision for staged surgical intervention highlights the multidisciplinary approach required in managing central neurocytoma, involving neurosurgery, radiology, and critical care teams [10,11]. In this case, the postoperative course was complicated by difficulty maintaining oxygen saturation, necessitating a planned tracheostomy. While uncommon, respiratory complications following neurosurgical procedures can occur, emphasizing the importance of perioperative monitoring and management of airway patency. The need for tracheostomy underscores the potential for significant morbidity associated with central neurocytoma and its surgical management. Histopathological examination confirmed the diagnosis of central neurocytoma, further guiding postoperative management and prognosis. Central neurocytoma is typically classified as grade II or IV according to the WHO classification of central nervous system tumors, with grade IV tumors associated with a higher risk of recurrence and malignant behavior [12].

## Conclusions

In conclusion, the presented case underscores the intricate nature of central neurocytoma, a rare intracranial tumor with diverse clinical presentations. Through this case, we have emphasized the significance of considering central neurocytoma in diagnosing patients exhibiting neurological deficits and hydrocephalus. A multidisciplinary collaboration involving neurosurgery, radiology, and critical care teams is essential for comprehensive patient management and favorable outcomes. Surgical resection remains the cornerstone of treatment, aiming to alleviate symptoms and prevent complications such as obstructive hydrocephalus. However, the postoperative course can be complicated by respiratory issues or other complications, highlighting the importance of vigilant perioperative monitoring and management. Histopathological examination is crucial in confirming the diagnosis and guiding further management strategies. This case underscores the need for continued research to enhance our understanding of central neurocytoma and refine treatment approaches for this challenging intracranial tumor.
